# A Pyroptosis-Related Gene Signature to Predict Patients' Prognosis and Immune Landscape in Liver Hepatocellular Carcinoma

**DOI:** 10.1155/2022/1258480

**Published:** 2022-02-16

**Authors:** Jiakun Wang, Zhihao Huang, Hongcheng Lu, Rongguiyi Zhang, Qian Feng, Aoxiao He

**Affiliations:** ^1^Department of General Surgery, The Second Affiliated Hospital of Nanchang University, Nanchang 330000, China; ^2^Department of Emergency, The Second Affiliated Hospital of Nanchang University, Nanchang 330000, China

## Abstract

**Background:**

Liver hepatocellular carcinoma (LIHC) is a malignance with high incidence and recurrence. Pyroptosis is a programed cell death pattern which both activates the effective immune response and causes cell damage. However, their potential applications of pyroptosis-related genes (PRGs) in the prognostic evaluation and immunotherapy of LIHC are still rarely discussed.

**Methods:**

Comprehensive bioinformatics analyses based on TCGA-LIHC dataset were performed in the current study.

**Results:**

A total of 33 PRGs were selected to perform the current study. Of these 33 PRGs, 26 PRGs with upregulation or downregulation in LIHC tissues were identified. We also summarized the related genetic mutation variation landscape. GO and KEGG pathway analysis demonstrated that these 26 PRGs were primarily associated with pyroptosis, positive regulation of interleukin-1 beta production, and NOD-like receptor signaling pathway. An unfavorable OS appeared in LIHC patients with high CASP3, CASP4, CASP6, CASP8, GPX4, GSDMA, GSDME, NLRP3, NLRP7, NOD1, NOD2, PLCG1, and SCAF11 expression and low NLRP6 expression. A prognostic signature constructed by the above 14 prognostic PRGs had moderate to high accuracy to predict LIHC patients' prognosis. And risk score was correlated with the expression of CASP6, CASP8, GPX4, GSDMA, GSDME, NLRP6, and NOD2. Of these 7 genes, CASP8 was identified as the core gene in PPI network. Moreover, lncRNA MIR17HG/hsa-miRNA-130b-3p/CASP8 regulatory axis in LIHC was also detected.

**Conclusions:**

The current study indicated the crucial role of PRGs in the prognostic evaluation of LIHC patients and their correlations with tumor microenvironment in LIHC.

## 1. Introduction

Globally, liver cancer accounted for over 800000 new cases and caused over 700000 cancer-related death in 2018. Of these new cases, 85% were diagnosed as liver hepatocellular carcinoma (LIHC), and over 40% were diagnosed at advanced stage [1]. LIHC is the most common subtype of liver cancer. Although some risk factors have been identified, including hepatitis B infection, liver cirrhosis, and alcoholic and nonalcoholic fatty liver diseases, and many approaches have been utilized in clinical practice, mainly including surgical resection, liver transplantation, chemotherapy, targeted drug treatment, and immunotherapy, the 5-year survival rate of LIHC in some developing countries is still only 18% [2–4]. And the minority of LIHC patients are eligible for these approaches due to high costs of treatment, serious drug adverse reactions, and multidrug resistance of tumor [5]. In a word, the current developments in the diagnosis, treatment, and prognostic evaluation of LIHC cannot meet the demand of patients. In the past 30 years, aberrant expression of genes in tumor tissues and their potential applications in clinical practice have been focused by clinicians. Thus, exploring potential gene signatures for therapeutic and prognostic assessment of LIHC is significant clinically.

Pyroptosis is one of programmed cell death patterns and both stimulates effective immune responses and causes tissue damage [6]. The process of pyroptosis was firstly described by Zychlinsky et al. in 1992; they revealed that death of macrophages infected by *Shigella flexneri* was dependent on CASP1 [7]. With the in-depth studies on the mechanisms of pyroptosis, increasing genes that regulate pyroptosis are identified, which mainly include AIM2, CASP1, CASP3, CASP4, CASP5, CASP6, CASP8, CASP9, ELANE, GPX4, GSDMA, GSDMB, GSDMC, GSDMD, GSDME, IL-18, IL-1B, IL-6, NLRC4, NLRP1, NLRP2, NLRP3, NLRP6, NLRP7, NOD1, NOD2, PJVK, PLCG1, PRKACA, PYCARD, SCAF11, TIRAP, and TNF [8–11]. And some studies had found that pyroptosis could influence the prognosis of cancer patients through controlling cancer cell proliferation, invasion, and metastasis [12, 13]. With the development of machine learning and big data technique, gene signature had been used to guide clinical evaluation of cancer patients. Previously, pyroptosis-related gene (PRG) signatures concerning lung adenocarcinoma, skin cutaneous melanoma, and glioblastoma had been established [14–16]. However, the potential clinical applications of pyroptosis-related gene signature in LIHC are still unclear.

With the rapid development of molecular biology and the enrichment of genomic data, comprehensive analysis on PRG signatures becomes feasible. In the present study, bioinformatic data was utilized to explore the expression profiles, prognostic performance, and related regulation axis of PRG signatures in LIHC. Moreover, we also explored the association between immune cell infiltration and PRG signatures in LIHC. The above findings may offer novel insights on the prognostic evaluation and therapy of LIHC.

## 2. Materials and Methods

### 2.1. Datasets

The genomic data of LIHC patients and related clinical data of these patients, including gender, age, tumor grade, and survival outcome, were downloaded from the Cancer Genome Atlas (TCGA) database on August 1, 2021. The TCGA-LIHC dataset (*N* = 374) was selected to perform the analyses. The data of copy number variation (CNV) and somatic mutation in LIHC were also extracted from TCGA database and UCSC Xena, respectively. Using R software V4.0.3, statistical analyses were performed. In addition, the *P* value cutoff was set as 0.05.

### 2.2. Identification of Differently Expressed PRGs

Based on previous studies [8–11], a total of 33 PRGs were summarized to perform our study, including AIM2, CASP1, CASP3, CASP4, CASP5, CASP6, CASP8, CASP9, ELANE, GPX4, GSDMA, GSDMB, GSDMC, GSDMD, GSDME, IL-18, IL-1B, IL-6, NLRC4, NLRP1, NLRP2, NLRP3, NLRP6, NLRP7, NOD1, NOD2, PJVK, PLCG1, PRKACA, PYCARD, SCAF11, TIRAP, and TNF. The list of these genes and corresponding full name is shown in Supplementary Table 1. The transcriptional levels of these 33 PRGs in LIHC tissues and normal liver tissues were visualized using TCGA-LIHC dataset by “reshape2” and “limma” packages in R software. Student's *t*-test was utilized in this analysis, and the expression data were standardized to transcripts per kilobase million (TPM) values before subsequent process.

### 2.3. Mutation Analysis and Functional Enrichment Analysis of PRGs

Mutation categories and mutation frequency of the 33 PRGs as well as their waterfall plots were constructed by “maftools” package in R. And the location of variation of these genes on 23 chromosomes was shown by “RCircos” package. Functional enrichment analysis, including Gene Ontology (GO) analysis and Kyoto Encyclopedia of Genes and Genomes (KEGG) pathway analysis, was performed to explore the potential molecular mechanisms and biological functions of target genes by Metascape (http://metascape.org/gp/index.html#/main/step1) [17]. Moreover, GO analysis included biological process (BP), cellular component (CC), and molecular function (MF) analysis.

### 2.4. Consensus Cluster Analysis

Setting the threshold as follows: iteration = 100 and resample rate = 80%, consensus cluster analysis was conducted by “ConsensusClusterPlus” package in R. “Survival” package was utilized to evaluate the difference of each cluster in LIHC. Next, the difference of clinical paraments in each cluster was explored by “pheatmap” package. Furthermore, the ImmuneScore, ESTIMATEScore, StromalScore, and abundance of immune cell in each cluster were calculated by the ESTIMATE algorithm [18]. The comparison of above indexes was visualized by the “vioplot” and “ggpubr” package.

### 2.5. Prognostic Analysis and Prognostic Model Construction of PRGs

The prognostic value of differently expressed PRGs was assessed by the “survival” package in R and was expressed as prognostic forest maps. The differently expressed PRGs with OS difference in low-expression and high-expression group were defined as prognostic PRGs. According to the above prognostic PRGs, LASSO Cox regression analysis in “glmnet” package was used to construct prognostic model. Those PRGs that constituted prognostic model were selected for further analyses. According to the median risk score, patients were divided into low- and high-risk group, and the OS curves of the two groups were compared. The predictive accuracy of this prognostic model was assessed by time ROC analysis. The univariate and multivariate Cox analysis was performed to excavate the influence of clinical factors, including age, gender, tumor grade, clinical stage and TNM stage, and risk score on the prognosis of LIHC patients. Subgroup analyses on the influence of clinical factors on the prognosis of low-risk and high-risk group were also performed. We were also interested in the difference of risk score in different subgroups, including age, gender, cluster, tumor grade, clinical stage, ImmuneScore, and TNM stage. Besides, the correlation between abundance of immune cells and risk score was also analyzed.

### 2.6. PPI Network Construction of PRGs in LIHC

The PRGs that made up risk score were selected via above analyses. Using STRING (https://string-db.org/), a PPI network was constructed to illustrate the association among these genes and seek for the core gene [19]. Further analyses were performed to indicate the potential mechanisms, biological functions, and applications of the core gene in LIHC.

### 2.7. Immune Infiltration, MSI, and TMB Analysis of CASP8 in LIHC

TIMER (http://timer.cistrome.org/) [20] is a bioinformatics platform that aims to illustrate the correlation between target gene and various immune cells. The “Gene” module was used to validate the correlation between the expression of CASP8 and the abundance of B cell, CD8+ T cell, CD4+ T cell, macrophage, neutrophil, and dendritic cell. Next, we investigated the correlation between somatic copy number alterations (SCNAs) of CASP8 and immune infiltration level of different immune cells by the “SCNA” module. Furthermore, tumor mutation burden (TMB) and microsatellite instability (MSI) analysis were used to assess the relation between the expression of CASP8 and TMB and MSI scores, respectively.

### 2.8. mRNA-miRNA-lncRNA Network Construction of CASP8 in LIHC

A mRNA-miRNA-lncRNA network was constructed for finding out the potential regulatory axis of CASP8 in LIHC. The miRNA targets binding to CASP8 in LIHC were excavated by miRDB (http://www.mirdb.org/) [21], miRWalk (http://mirwalk.umm.uni-heidelberg.de/) [22], and StarBase (https://starbase.sysu.edu.cn/starbase2/index.php) [23] database. The common ground in these three databases was identified as the most significantly connected miRNAs. Next, according to the selected miRNAs, the lncRNA targets interacting to miRNAs were explored by StarBase and LncBase (https://diana.e-ce.uth.gr/lncbasev3) [24], and the association between the miRNAs and the lncRNAs was visualized. The expression level and prognostic performance of the targeted miRNAs and lncRNAs were also evaluated using TCGA-LIHC dataset.

## 3. Results

### 3.1. The mRNA Levels and Genetic Variation Landscape of PRGs in LIHC

**Figure 1:**
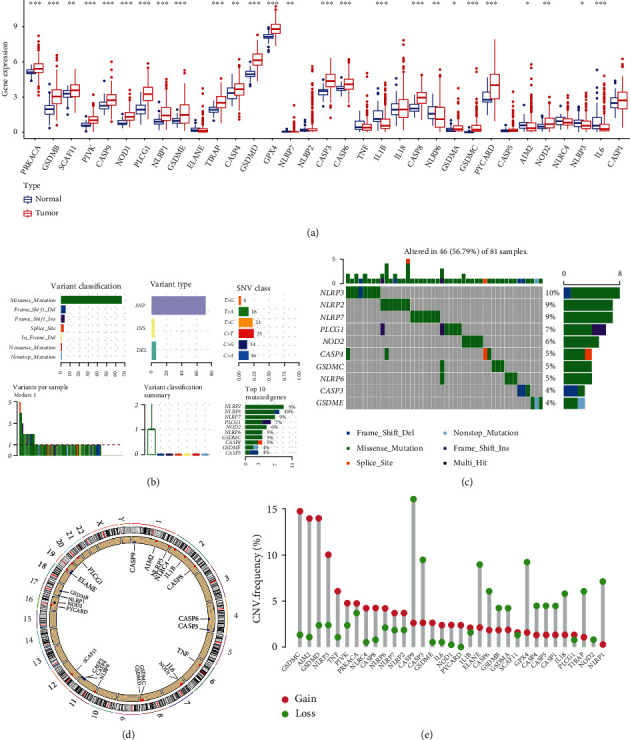
Landscape of genetic variation and expression of PRGs in LIHC. (a) The mRNA levels of PRGs in LIHC and normal liver tissues. (b, c) The mutation frequency and classification of PRGs in LIHC. (d) The location of CNV alteration of PRGs on 23 chromosomes in LIHC. (e) The CNV variation frequency of PRGs in LIHC. The height of the column represented the alteration frequency. Note: ^∗^*P* < 0.05, ^∗∗^*P* < 0.01, ^∗∗∗^*P* < 0.001.

According to the RNA-sequencing data from TCGA-LIHC dataset, the mRNA levels of 33 pyroptosis-related genes in LIHC tissues and normal liver tissues were shown in Figure 1(a). Compared to normal liver tissues, the transcriptional levels of PRKACA, GSDMB, SCAF11, PJVK, CASP9, NOD1, PLCG1, NLRP1, GSDME, TIRAP, CASP4, GSDMD, GPX4, CASP3, CASP6, CASP8, GSDMA, GSDMC, PYCARD, and NOD2 were elevated in LIHC tissues, while the mRNA levels of NLRP7, IL-1B, NLRP6, AIM2, NLRP3, and IL-6 were decreased in LIHC tissues. Moreover, no significant difference was detected between the expression levels of ELANE, NLRP2, TNF, IL-18, CASP5, NLRC4, and CASP1 in LIHC tissues and those in normal liver tissues. We were also interested in the copy number variations and somatic variations of 33 pyroptosis-related genes in LIHC. As shown in Figures 1(b) and 1(c), genetic alterations were detected in 46 (56.79%) of 81 LIHC samples. The categories of genetic alteration included missense mutation, frame-shift deletion, frame-shift insertion, splice-site variation, in-frame deletion, nonsense mutation, and nonstop mutation. And the most common variant classification was missense mutation. In addition, SNP was the most common variant type, and C>T ranked top SNV class. Of these 33 PRGs, NLRP3 was the gene with the highest mutation frequency. The location of the CNV variations on chromosomes was also presented in Figure 1(d). The CNV variation frequency of the 33 PRGs in LIHC was also summarized (Figure 1(e)). Copy number deletion was detected in CASP3, CASP9, ELANE, CASP6, GSDMB, GSDMA, GPX4, CASP1, CASP4, CASP5, IL-18, TIRAP, NOD2, and NLRP1, while copy number amplification was detected in AIM2, GSDMD, GSDMC, NLRP3, TNF, PJVK, PRKACA, NLRC4, CASP8, NLRP6, NLRP7, NLRP2, GSDME, IL-6, NOD1, PYCARD, IL-1B, SCAF11, and PLCG1.

### 3.2. Functional Enrichment Analysis of PRGs in LIHC

**Figure 2:**
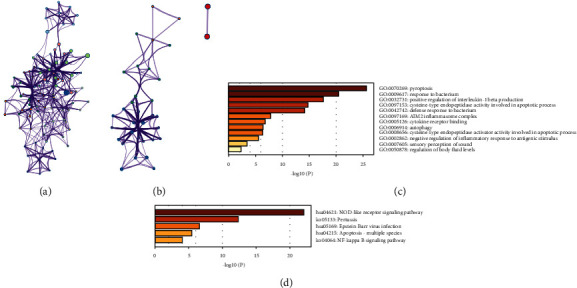
Functional enrichment analysis of 26 upregulated or downregulated PRGs in LIHC. (a) The relationship network of the 26 PRGs in GO analysis. (b) The relationship network of the 26 PRGs in KEGG pathway analysis. (c) The bar plot of GO analysis, including BP, CC, and MF analysis. (d) The bar plot of KEGG pathway analysis.

The 26 PRGs with aberrant expression in LIHC tissues were selected to perform GO and KEGG pathway analysis. To capture the relationship between the enriched terms, the networks of GO analysis and KEGG pathway analysis were presented in Figures 2(a) and 2(b), respectively. For further visualizing the potential biological functions and molecular mechanisms of these 26 genes in LIHC tissues, the results of GO analysis (Figure 2(c)) and KEGG pathways analysis (Figure 2(d)) were shown in bar plots. The results of GO analysis revealed these 26 PRGs mainly participated in pyroptosis, response to bacterium, positive regulation of interleukin-1 beta production, and cysteine-type endopeptidase activity involved in apoptotic process. The results of KEGG pathway analysis indicated these 26 PRGs were mainly associated with NOD-like receptor signaling pathway, pertussis, Epstein-Barr virus infection, apoptosis, and NF-kappa B signaling pathway.

### 3.3. Consensus Clustering Categorized LIHC Patients

**Figure 3:**
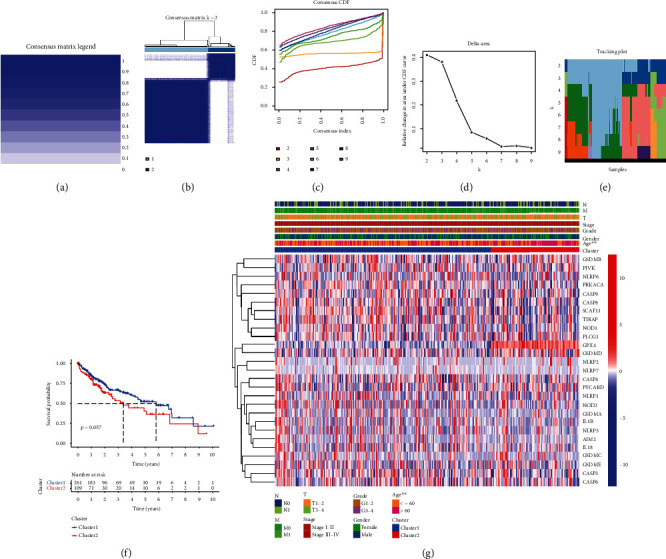
Consensus cluster analysis of the 26 PRGs in LIHC. The consensus matrix legend (a) and consensus clustering matrix (*K* = 2) (b) in this consensus cluster analysis. The consensus clustering CDF (c), relative change in area under CDF curve (d), and tracking plot (e) for *K* = 2 to 9. (f) Comparison of OS plot in cluster 1 and cluster 2. (g) Heatmap revealed the influence of clinical parameters on the expression of cluster 1 and cluster 2. Note: ^∗∗^*P* < 0.01.

Based on the above 26 PRGs with aberrant expression, we consensus clustering categorized LIHC patients. According to the similarity displayed by expression level and the proportion of ambiguous clustering measure, optimal clustering stability appeared when *k* value = 2 (Figures 3(a) and 3(b)). The cumulative distribution function, increment in the AUC, and the tracking plot of subgroup *k* value = 2 to 9 were visualized in Figures 3(c)–3(e), respectively. Next, we analyzed the prognostic performance of cluster 1 and cluster 2 (Figure 3(f)). There is no statistical difference between the overall survival curve of cluster1 and that of cluster 2 (*P* value = 0.057). Moreover, we also studied the association between clinical parameters and gene expression of cluster 1 and cluster 2 and found that age (>60 years or not) is a factor that influence the gene expression of cluster 1 and cluster 2 (Figure 3(g), *P* value < 0.01).

### 3.4. The Correlation between Cluster and Immune Cell Infiltration

**Figure 4:**
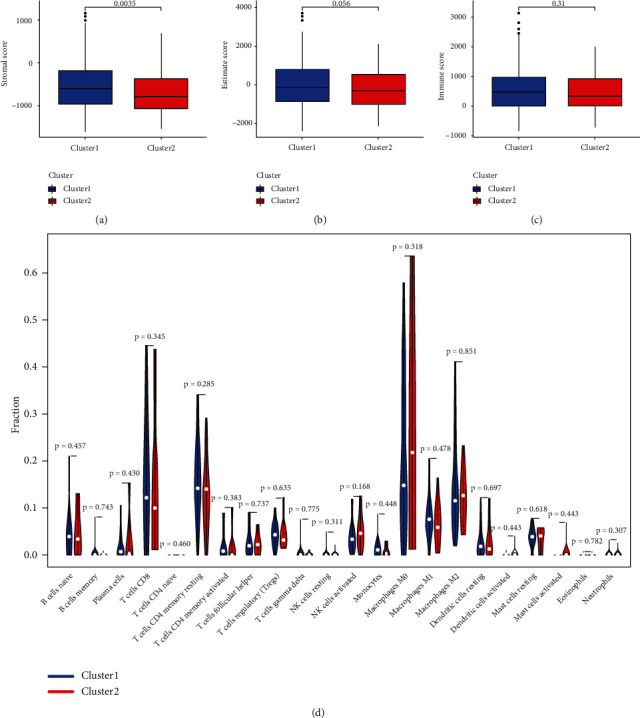
The correlation between cluster and immune cell infiltration. (a) Cluster 1 had a higher StromalScore than cluster 1. No statistical difference was found in ESTIMATEScore (b) and ImmuneScore (c) between cluster 1 and cluster 2. (d) The infiltrating landscape of 22 immune cells in the two clusters.

To investigate the difference of the 2 clusters in tumor microenvironment of LIHC, we explored the relationship between immune cell infiltration and cluster. The StromalScore in cluster 1 was significantly higher than that in cluster 2 (Figure 4(a), *P* value = 0.0035). However, there was no significant difference in the ESTIMATEScore (Figure 4(b), *P* value = 0.056) and ImmuneScore (Figure 4(c), *P* value = 0.31) between the two clusters. We next explored the difference between abundance of different immune cells in the two clusters. Unexpectedly, there was no significant difference in the abundance of naive B cells (*P* value = 0.457), memory B cells (*P* value = 0.743), plasma cells (*P* value = 0.430), CD8+ T cells (*P* value = 0.345), naive CD4+ T cells (*P* value = 0.460), resting memory CD4+T cells (*P* value = 0.285), activated memory CD4+T cells (*P* value = 0.383), follicular helper T cells (*P* value = 0.737), Tregs (*P* value = 0.635), gamma delta T cells (*P* value = 0.775), resting NK cells (*P* value = 0.311), activated NK cells (*P* value = 0.168), monocytes (*P* value = 0.448), M0 macrophages (*P* value = 0.318), M1 macrophages (*P* value = 0.478), M2 macrophages (*P* value = 0.851), resting dendritic cells (*P* value = 0.697), activated dendritic cells (*P* value = 0.443), resting mast cells (*P* value = 0.618), activated mast cells (*P* value = 0.443), eosinophils (*P* value = 0.782) and neutrophils (*P* value = 0.307) between the two clusters (Figure 4(d)).

### 3.5. Prognostic Performance and Prognostic Model Construction of PRGs in LIHC

**Figure 5:**
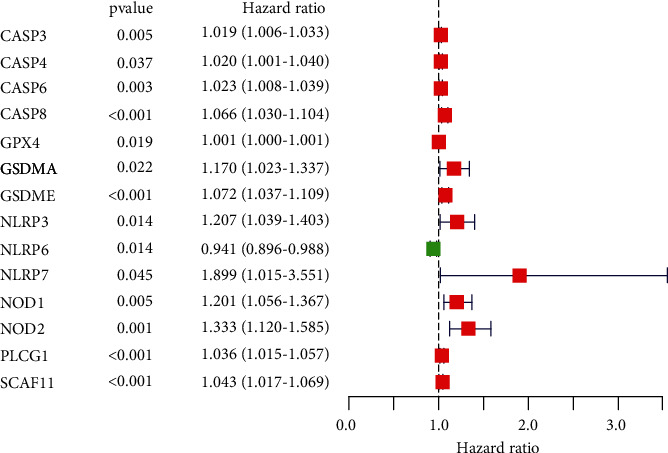
The prognostic performance of the 26 PRGs in LIHC. LIHC patients with high CASP3, CASP4, CASP6, CASP8, GPX4, GSDMA, GSDME, NLRP3, NLRP7, NOD1, NOD2, PLCG1, and SCAF1 had worse OS than those with low expression of these genes, while LIHC patients with high NLRP6 expression had better OS than those with low NLRP6 expression. A total of 14 PRGs were identified as prognostic PRGs.

**Figure 6:**
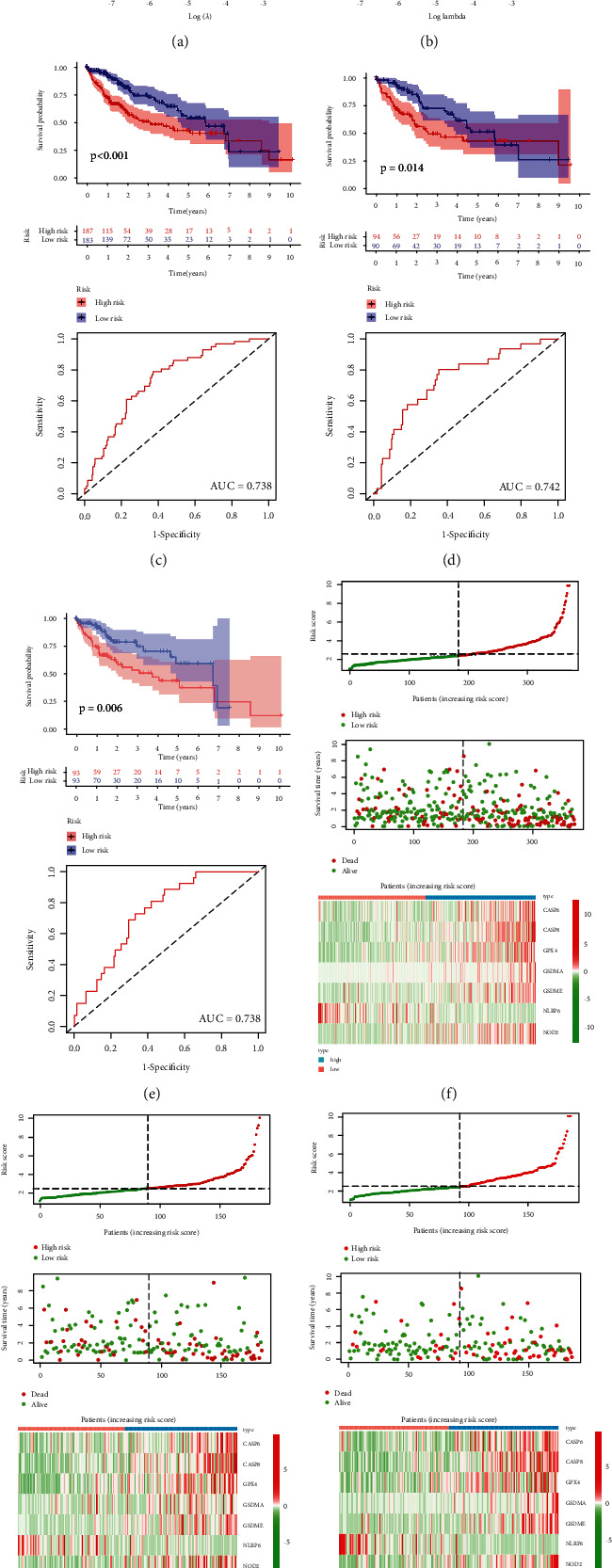
Prognostic model constructed by the 14 prognostic PRGs. The partial likelihood deviance (a) and coefficients (b) of prognostic model. The OS plots and corresponding ROC curves of high-risk group and low-risk group in all LIHC cohort (c), in test cohort (d), and training cohort (e). Risk score distribution, patients' survival status, and expression of 7 PRGS associated with risk score in all LIHC cohort (f), in test cohort (g), and training cohort (h).

**Figure 7:**
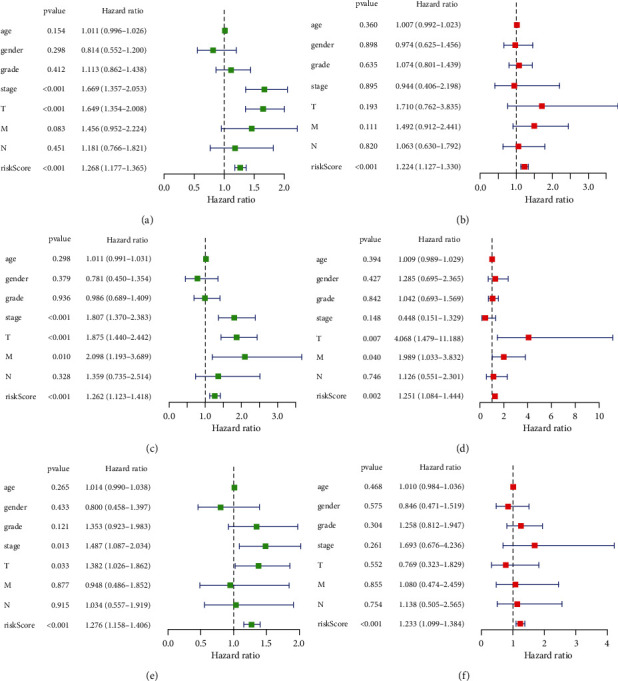
Univariate and multivariate Cox regression analysis considering age, gender, tumor grade, clinical stage, T stage, N stage, M stage, and risk score in all LIHC cohort (a, b), in test cohort (c, d), and training cohort (e, f).

**Figure 8:**
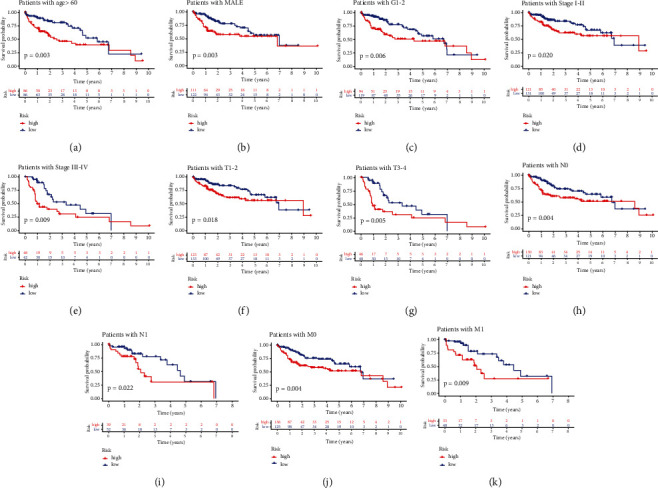
The OS curves of high-risk group and low-risk group based on the subgroups of age (a), gender (b), tumor grade (c), clinical stage (d, e), T stage (f, g), N stage (h, i), and M stage (j, k).

We also investigated the prognostic performance of above 26 PRGs. LIHC patients with high CASP3 (*P* value = 0.005), CASP4 (*P* value = 0.037), CASP6 (*P* value = 0.003), CASP8 (*P* value < 0.001), GPX4 (*P* value = 0.019), GSDMA (*P* value = 0.022), GSDME (*P* value < 0.001), NLRP3 (*P* value = 0.014), NLRP7 (*P* value = 0.045), NOD1 (*P* value = 0.005), NOD2 (*P* value = 0.001), PLCG1 (*P* value < 0.001), and SCAF11 (*P* value < 0.001) had worse OS than those with low expression of these genes, while LIHC patients with high NLRP6 expression had better OS than those with low NLRP6 expression (Figure 5, *P* value = 0.014). No significant difference was found in OS between LIHC patients with high and low PRKACA, GSDMB, PJVK, CASP9, NLRP1, TIRAP, GSDMD, GSDMC, PYCARD, IL-1B, AIM2, and IL-6 expression (*P* value > 0.05). Thus, a total of 14 genes were consider as prognostic PRGs and were selected for further analyses. For better predicting the prognosis of LIHC patients, the 14 prognostic PRGs were selected to construct prognostic model by LASSO Cox analysis. The partial likelihood deviance and coefficients of prognostic model were presented in Figures 6(a) and 6(b). Risk score = (0.0024)∗CASP6 expression + (0.0468)∗CASP8 expression + (0.0011)∗GPX4 expression + (0.0821)∗GSDMA expression + (0.0132)∗GSDME expression + (−0.0240)∗NLRP6 expression + (0.0830)∗NOD2 expression. LIHC patients were divided into high-risk and low-risk group according to the risk score. The OS plots of all LIHC cohort (*P* value < 0.001, Figure 6(c)), test cohort (*P* value = 0.014, Figure 6(d)), and training cohort (*P* value = 0.006, Figure 6(e)) revealed that high-risk group had worse OS than low-risk group with AUC of 0.738, 0.742, and 0.738, respectively. Figures 6(f)–6(h) illustrated the risk score distribution, patients' survival status, and gene expression level of low-risk and high-risk group in all LIHC cohort, test cohort, and training cohort, respectively. Next, univariate and multivariate analyses were performed to find out the potential factors that influenced the prognosis of LIHC patients. Evidently, risk score was considered as the independent factor affecting LIHC patients' prognosis in all LIHC cohort (Figures 7(a) and 7(b)), test cohort (Figures 7(c) and 7(d)), and training cohort (Figures 7(e) and 7(f)). Further, we also explored whether the prognostic model was suitable for LIHC patients with different clinical features. In the subgroup of age > 60 years (Figure 8(a), *P* value = 0.003), male patients (Figure 8(b), *P* value = 0.003), patients with grades 1-2 (Figure 8(c), *P* value = 0.006), patients with stages I-II (Figure 8(d), *P* value = 0.020), patients with stages III-IV (Figure 8(e), *P* value = 0.009), patients with T1-2 (Figure 8(f), *P* value = 0.018), patients with T3-4 (Figure 8(g), *P* value = 0.005), patients with N0 (Figure 8(h), *P* value = 0.004), patients with N1 (Figure 8(i), *P* value = 0.022), patients with M0 (Figure 8(j), *P* value = 0.004), and patients with M1 (Figure 8(k), *P* value = 0.009), high-risk group showed worse OS than low-risk group.

### 3.6. Risk Score Associated with Clinical Parameters and Immune Cell Infiltration

**Figure 9:**
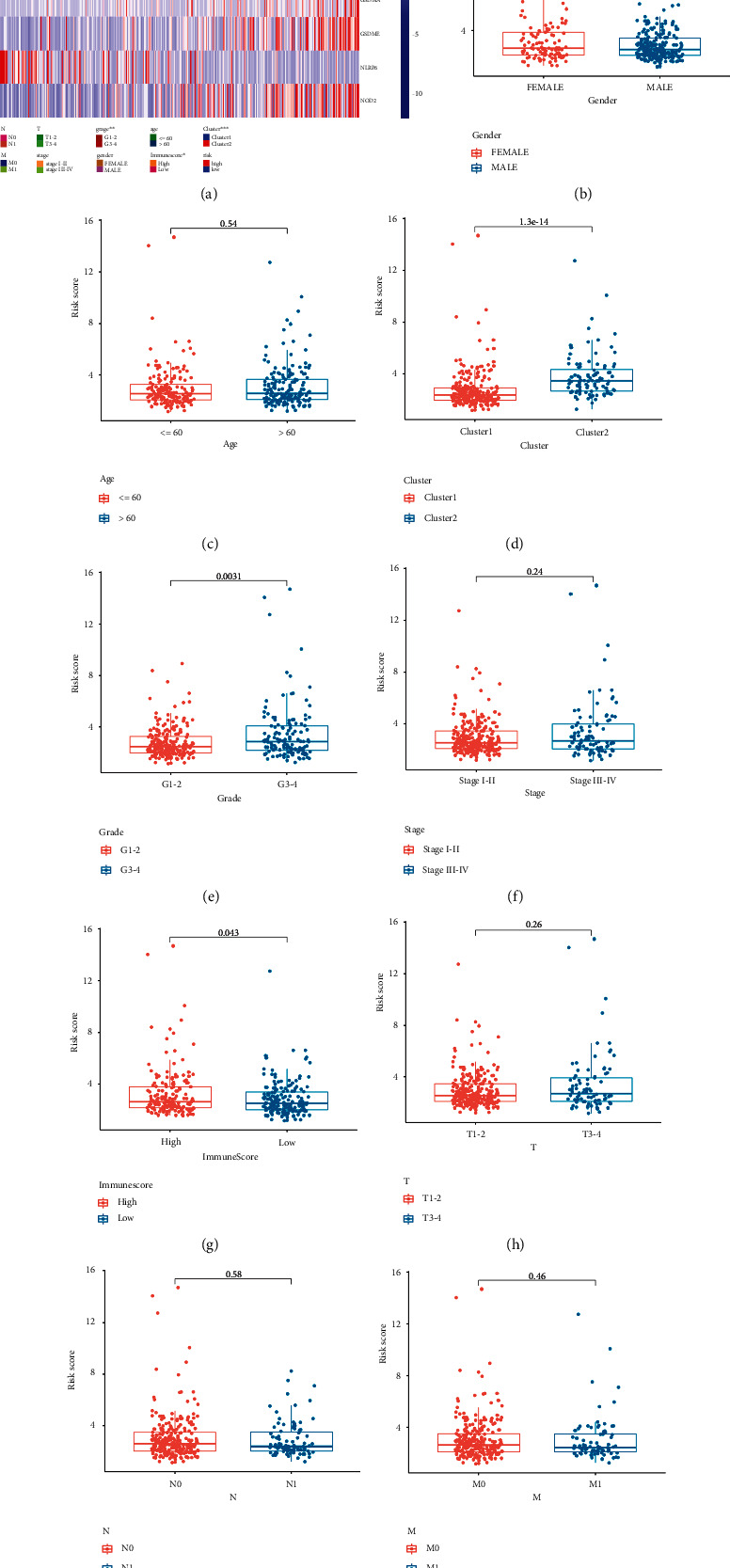
Risk score associated with clinical features in LIHC. (a) The heatmap revealed the difference of clinical characteristics between high-risk group and low-risk group. The difference of risk score in subgroups of gender (b), age (c), cluster (d), tumor grade (e), clinical stage (f), ImmuneScore (g), T stage (h), N stage (i), and M stage (j). Note: ^∗^*P* < 0.05, ^∗∗^*P* < 0.01, ^∗∗∗^*P* < 0.001.

We also focused on the influence of clinical parameters on risk score. The expression of the genes that constituted risk score and distribution of clinical characteristics in low-risk and high-risk group is summarized in Figure 9(a). Compared with cluster 1, LIHC patients in cluster 2 had higher risk score (Figure 9(d), *P* value = 1.3*E*-14). LIHC patients with grades III-IV showed higher risk score than those with grades I-II (Figure 9(e), *P* value = 0.0031), and LIHC patients with high ImmuneScore showed higher risk score than those with low ImmuneScore (Figure 9(g), *P* value = 0.043). However, no significant difference was detected in the subgroups of gender, age, clinical stage, T stage, N stage, and M stage (Figures 9(b), 9(c), 9(f), and 9(h)–9(j), all *P* value > 0.05). In addition, the association between immune cell infiltration and risk score was also explored. However, only the correlation between abundance of activated memory CD4+ T cells and risk score was detected with a *P* value of 0.27 (Supplementary Figure 1(a)). Thus, more studies are urgently needed to fill the gaps.

### 3.7. Immune Cell Infiltration, MSI, and TMB Analysis of CASP8 in LIHC

**Figure 10:**
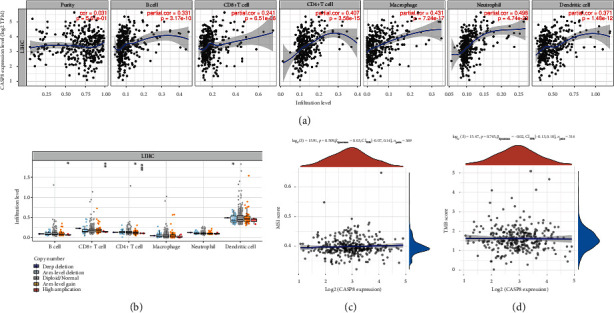
Immune infiltration, TMB, and MSI analysis of CASP8 in LIHC. (a) The expression level of CASP8 was positively correlated with abundance of B cell, CD8+ T cell, CD4+ T cell, macrophage, neutrophil, and dendritic cell. (b) The association between infiltration levels of B cell, CD8+ T cell, CD4+ T cell, macrophage, neutrophil and dendritic cell, and SCNAs. (c) The correlation between MSI score and expression level of CASP8 in LIHC. (d) The correlation between TMB score and expression level of CASP8 in LIHC. Note: ^∗^*P* < 0.05, ^∗∗^*P* < 0.01, ^∗∗∗^*P* < 0.001.

We also constructed PPI network to visualize the association among the genes that constituted risk score (Supplementary Figure 1(b)), which indicated that CASP8 was the core gene in the PPI network. Therefore, further analyses focusing on CASP8 in LIHC were performed. Tumor-infiltrating immune cells regulate occurrence and progression of tumor through extremely complicated mechanisms. Thus, we attempted to illustrate the correlation between immune cell infiltration and CASP8 expression level in LIHC tissues. The expression level of CASP8 was positively correlated with abundance of B cell (cor = 0.331, *P* value = 3.17*E*-10), CD8+ T cell (cor = 0.241, *P* value = 6.51*E*-06), CD4+ T cell (cor = 0.407, *P* value = 3.58*E*-15), macrophage (cor = 0.431, *P* value = 7.24*E*-17), neutrophil (cor = 0.498, *P* value = 4.74*E*-23), and dendritic cell (cor = 0.371, *P* value = 1.48*E*-12) (Figure 10(a)). Next, we explored the correlation between SCNAs of CASP8 and the infiltration levels of six immune cells in LIHC tissues. The infiltration levels of B cell, CD8+ T cell, CD4+ T cell, macrophage, neutrophil, and dendritic cell were generally decreased when SCNAs appeared (Figure 10(b)). Tumor mutation burden (TMB) and microsatellite instability (MSI) could assist clinicians to predict the efficacy of cancer immunotherapy. However, no significant correlation was detected between MSI score and CASP8 expression (Figure 10(c), *P* value = 0.509). In TMB analysis, similar result was presented in Figure 10(d) (*P* value = 0.745).

### 3.8. Construction of a mRNA-miRNA-lncRNA Network

**Figure 11:**
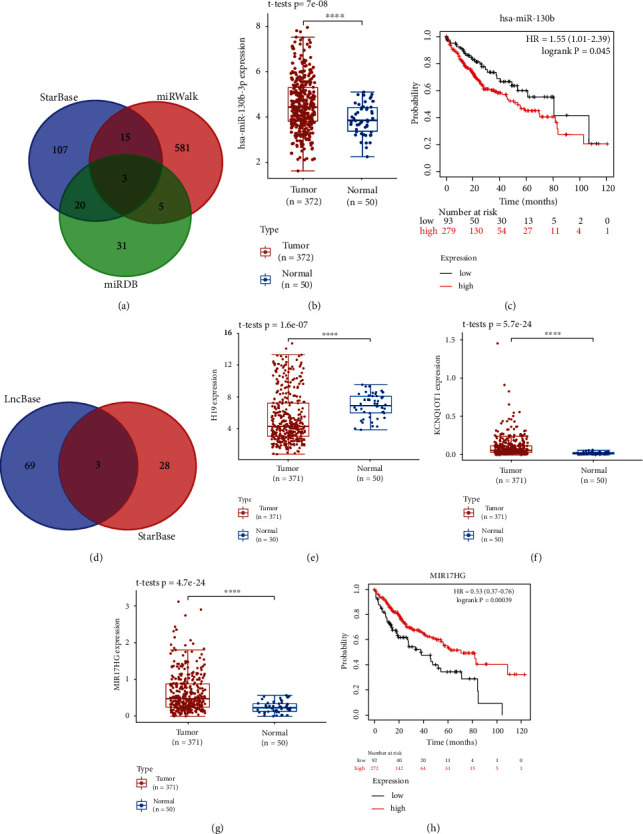
Construction of a mRNA-miRNA-LncRNA network. (a) Results of miRNA target predicted by miRWalk, miRDB, and StarBase. The expression level (b) and prognostic value (c) of miRNA-130b-3p in LIHC. (d) Results of lncRNA targets predicted by lncBase and StarBase. The expression level of lncRNA H19 (e), KCNQ1OT1 (f), and MIR17HG (g) in LIHC. (h) The prognostic value of MIR17HG in LIHC. Note: ^∗∗∗∗^*P* < 0.0001.

The above analyses indicated that CASP8 was correlated with immune cell infiltration in LIHC. And CASP8 acted as the core gene in the PPI network constructed by PRGs associated with risk score. Thus, CASP8 was selected to construct a mRNA-miRNA-lncRNA network, which might uncover the potential CASP8-related regulatory axis in LIHC. First of all, CASP8 targeting miRNAs in miRDB, StarBase, and miRWalk database were compared to identify the common ground, which included hsa-miR-519a-3p, hsa-miR-105-5p, and hsa-miR-130b-3p (Figure 11(a)). Next, we explored the expression and prognostic value of these three genes in LIHC. The result indicated that only hsa-miRNA-130b-3p was differentially expressed in tumors and is significantly associated with patient prognosis. To be more specific, the expression level of hsa-miRNA-130b-3p was significantly elevated in LIHC tissues compared with normal liver tissues (Figure 11(b), *P* value = 7*E*-08), and LIHC patients with high hsa-miRNA-130b-3p expression had worse OS than those with low hsa-miRNA-130b-3p expression (Figure 11(c), *P* value = 0.045). Thus, hsa-miRNA-130b-3p was considered as the most promising miRNA target of CASP8 in LIHC. The upstream lncRNA targets interacting to the promising miRNA targets were also explored via LncBase and StarBase database. A total of 3 lncRNA targets were identified based on the data in above databases, including H19, KCNQ1OT1, and MIR17HG (Figure 11(d)). The expression and prognostic performance of these three genes in LIHC were also analyzed. Compared to normal liver tissues, the expression level of H19 was decreased in LIHC tissues (Figure 11(e), *P* value = 1.6*E*-07), while the expression level of KCNQ1OT1 (Figure 11(f), *P* value = 5.7*E*-24) and MIR17HG (Figure 11(g), *P* value = 4.7*E*-24) were upregulated in LIHC tissues. Nevertheless, only MIR17HG was correlated with the prognosis of LIHC patients, which showed a better OS in high expression group (Figure 11(h), *P* value = 0.00039). Thus, MIR17HG was considered as the most promising lncRNA target of CASP8 in LIHC. According the above results, lncRNA MIR17HG/hsa-miRNA-130b-3p/CASP8 regulatory axis was detected and might influence the occurrence and progression of LIHC.

## 4. Discussion

Pyroptosis is defined as a type of programed cell death that is dependent on the gasdermin protein family, and its occurrence is often as a result of activation of inflammatory CASP protein [25]. With the development of molecular biology, genetics, and medicine, the biological functions, mechanisms, and potential applications of pyroptosis are gradually uncovered. In the field of cancer research, some studies have summarized the important role of pyroptosis, which indicates that pyroptosis may influence the occurrence and progression of cancer in all stage [26, 27]. And pyroptosis is considered as a promising mechanism in the cancer treatment due to the antiapoptotic effect of cancer cells [28]. However, the topic focusing on the role of PRGs in the prognosis and immune microenvironment of tumor is limited. Thus, we performed this bioinformatics analysis to systematically enucleate the expression, prognostic performance, and the effects on the immune microenvironment of PRGs in LIHC.

We firstly explored the expression level of 33 PRGs in LIHC tissues and normal liver tissues. Of these 33 PRGs, 26 genes with aberrant expression in LIHC tissues were selected to perform functional enrichment analysis and consensus cluster analysis. Programed cell death pathway, mainly including apoptosis and pyroptosis, protects mammals from infection. Outer membrane vesicles of Gram-negative bacteria delivered various bacterial molecules to host cells; outer membrane vesicle-associated molecules participated in the activation of apoptosis and pyroptosis [29]. CASP1 could cleave inactive IL-1 family to generate mature IL-1 family, such as IL-1*β* and IL-18, and GSDMD pore could mediate the release of IL-1 via electrostatic filtering [30]. He et al. reported that gene deletion of GSDMD interdicted the occurrence of pyroptosis and the secretion of IL-1*β* [31]. And the process of pyroptosis in various human cancer types was associated with NLRP3-related signaling pathways and NF-*κ*B signaling pathway [12, 32–34]. To some extent, the above studies supported the result of functional enrichment analysis. However, more studies are required to validate the biological functions and potential mechanisms of PRGs in LIHC.

Prognostic model was widely utilized in prognostic evaluation of cancer patients, which provided epidemiological evidence for clinicians. In the current study, a total of 14 prognostic PRGs were enrolled to build up a prognostic model. Through further screening, the risk score consisted of the seven PRGs, including CASP6, CASP8, GPX4, GSDMA, GSDME, NLRP6, and NOD2. And the LIHC patients in high-risk score group generally showed worse OS compared to low-risk score group. Other three bioinformatics analyses, respectively, constructed prognostic model to explore the correlation between prognosis of lung adenocarcinoma, skin cutaneous melanoma and glioblastoma, and PRGs, which indicated a worse prognosis in high-risk group [14–16]. The above results were similar with ours; nevertheless, large-sample multicenter studies are urgently needed to validate these results.

In the current study, CASP8 was detected as the core gene in the PPI network constructed by PRGs associated with risk score. Thus, we were interested on the role of CASP8 in LIHC. Previous studies reported that CASP8 not only induced apoptosis of LIHC cells but also had a nonapoptotic function in proliferation-related DNA damage response through CASP8/RIPK1/FADD/cFLIP complex in LIHC cells [35]. And Koschny et al. found that a stronger nuclear stain of CASP8 in LIHC cells and high nuclear CASP8 stain was associated with unfavorable prognosis after surgery and high tumor cell proliferation [36]. Thus, to find out the balance point of CASP8 in anticancer function and tumor-promoting action is of clinical significance.

A potential mRNA-miRNA-lncRNA regulatory axis in LIHC, lncRNA MIR17HG/hsa-miRNA-130b-3p/CASP8 regulatory axis, was also detected in the present study. MIR17HG participated in the generation of miRNA-17-92 cluster, which included miRNA-17, miRNA-18a, miRNA-19a, miRNA-20a, miRNA-19b, and miRNA-92a [37]. These six downstream miRNAs were detected increased expression levels in LIHC tissues and could promote invasion and proliferation of LIHC proliferation [38]. Wang et al. reported that miRNA-130b was upregulated in LIHC tissues in comparison with normal liver tissues, and high miRNA-130b was correlated with decreased survival, and overexpression of miR-130b promoted proliferation and metastasis of LIHC cells [39, 40]. The above studies revealed the potential of this regulatory axis in LIHC cells. However, related in vivo and in vitro researches are urgently required.

Some limitations still exist in the current study. Firstly, most of the analyses in the current study are conducted at the transcription level; some results may not apply to the studies based on the protein level. Secondly, related fundamental and clinical studies that focus on the clinical value and molecule mechanisms of PRGs in LIHC are rare. Third, genetic background and etiology of LIHC patients are influenced by many factors, such as patients' race, patients' gender, and patients' age. Thus, more in-depth studies are necessary to validate the results.

## 5. Conclusion

In summary, the present study built up a pyroptosis-related gene signature to predict LIHC patients' prognosis and their correlations with immune infiltration, which indicated that pyroptosis-related genes were of great significance in the LIHC patients' prognosis and microenvironment of LIHC. However, in-depth studies are needed to validate our results.

## Data Availability

The data used to support the findings of this study are available from the corresponding author upon request.

## Supplementary Materials

Supplementary 1 Supplementary Figure 1: immune cell infiltration analysis and PPI network concerning risk score. (a) The correlation between risk score and immune cell infiltration. (b) PPI network constructed by 7 PRGs associated with risk score.

Supplementary 2 Supplementary Table 1: the full names of 33 pyroptosis-related genes.
