# Supplementary figures and images for: A Pyroptosis-Related Gene Signature to Predict Patients' Prognosis and Immune Landscape in Liver Hepatocellular Carcinoma
Source: Comput Math Methods Med. 2022 Feb 16;2022:1258480. doi: 10.1155/2022/1258480 (PMC8886769; doi:10.1155/2022/1258480)

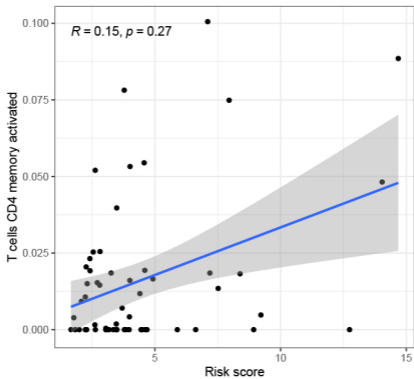

(a)

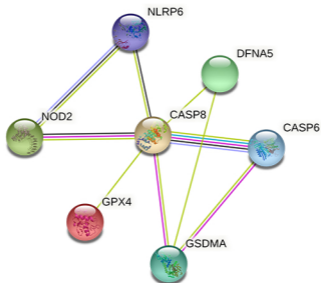

(b)

Supplement: Supplementary 1 — Supplementary Figure 1: immune cell infiltration analysis and PPI network concerning risk score. (a) The correlation between risk score and immune cell infiltration. (b) PPI network constructed by 7 PRGs associated with risk score. [file 1258480.f1.pdf]
