# Supplementary material for: A Pyroptosis-Related Gene Signature to Predict Patients' Prognosis and Immune Landscape in Liver Hepatocellular Carcinoma
Source: Comput Math Methods Med. 2022 Feb 16;2022:1258480. doi: 10.1155/2022/1258480 (PMC8886769; doi:10.1155/2022/1258480)
Supplement: Supplementary 2 — Supplementary Table 1: the full names of 33 pyroptosis-related genes. [file 1258480.f2.docx]

Supplementary Table 1. The full names of 33 pyroptosis-related genes.

| Genes | Full-names |
| --- | --- |
| AIM2 | Absent in melanoma 2 |
| CASP1 | cysteine-aspartic acid protease-1 |
| CASP3 | cysteine-aspartic acid protease-3 |
| CASP4 | cysteine-aspartic acid protease-4 |
| CASP5 | cysteine-aspartic acid protease-5 |
| CASP6 | cysteine-aspartic acid protease-6 |
| CASP8 | cysteine-aspartic acid protease-8 |
| CASP9 | cysteine-aspartic acid protease-9 |
| ELANE | elastase, neutrophil expressed |
| GPX4 | glutathione peroxidase 4 |
| GSDMA | gasdermin A |
| GSDMB | gasdermin B |
| GSDMC | gasdermin C |
| GSDMD | gasdermin D |
| GSDME | gasdermin E |
| IL18 | interleukin 18 |
| IL1B | interleukin 1 beta |
| IL6 | interleukin 6 |
| NLRC4 | NLR family CARD domain containing 4 |
| NLRP1 | NLR family pyrin domain containing 1 |
| NLRP2 | NLR family pyrin domain containing 2 |
| NLRP3 | NLR family pyrin domain containing 3 |
| NLRP6 | NLR family pyrin domain containing 6 |
| NLRP7 | NLR family pyrin domain containing 7 |
| NOD1 | nucleotide binding oligomerization domain containing 1 |
| NOD2 | nucleotide binding oligomerization domain containing 2 |
| PJVK | pejvakin/deafness, autosomal recessive 59 |
| PLCG1 | phospholipase C gamma 1 |
| PRKACA | protein kinase cAMP-activated catalytic subunit alpha |
| PYCARD | PYD and CARD domain containing |
| SCAF11 | SR-related CTD associated factor 11 |
| TIRAP | TIR domain containing adaptor protein |
| TNF | tumor necrosis factor |
